# Development of DNA Pair Biosensor for Quantization of Nuclear Factor Kappa B

**DOI:** 10.3390/bios8040126

**Published:** 2018-12-10

**Authors:** Zhaohui Wang, Pak Kin Wong

**Affiliations:** 1 Department of Electrical Engineering and Computer Science, Texas A&M University-Kingsville, Kingsville, TX 78363, USA; 2 Department of Aerospace & Mechanical Engineering, University of Arizona, Tucson, AZ 85721, USA; pak@email.arizona.edu or pxw28@psu.edu; 3 Biomedical Engineering and Mechanical Engineering, Department of Surgery, College of Medicine, Pennsylvania State University, University Park, PA 16802, USA

**Keywords:** biosensor, FRET, nuclear factor, DNA, fluorophore

## Abstract

Nuclear factor kappa B (NF-κB), regulating the expression of several genes that mediate the inflammatory responses and cell proliferation, is one of the therapeutic targets for chronic inflammatory disease and cancer. A novel molecular binding scheme for the detection of NF-κB was investigated for its affinity to Ig-κB DNA composed by dye and quencher fluorophores, and this specificity is confirmed by competing with the DNA sequence that is complementary to the Ig-κB DNA. We create a normalization equation to remove the negative effects from the various initial fluorophore concentrations and the background noise. We also found that a periodic shaking at a frequency could help to stabilize the DNA–protein binding. The calibration experiment, using purified p50 (NF-κB), shows that this molecular probe biosensor has a detection limit on the order of nanomolar. The limit of detection is determined by the binding performance of dye and quencher oligonucleotides, and only a small portion of probes are stabilized by DNA-binding protein NF-κB. The specificity experiment also shows that p50/p65 heterodimer has the highest affinity for Ig-κB DNA; p65 homodimer binds with intermediate affinity, whereas p50 shows the lowest binding affinity, and Ig-κB DNA is not sensitive to BSA (bovine albumin serum). The experiment of HeLa nuclear extract shows that TNF-α stimulated HeLa nuclear extract has higher affinity to Ig-κB DNA than non-TNF-stimulated HeLa nuclear extract (4-h serum response). Therefore, the molecular binding scheme provides a rapid, quantitative, high throughput, and automated measurement of the DNA-binding protein NF-κB at low cost, which is beneficial for automated drug screening systems.

## 1. Introduction

Nuclear factor kappa B (NF-κB) regulates the expression of several genes that mediate the inflammatory responses and cell proliferation and is one of the therapeutic targets for chronic inflammatory disease and cancer [1,2,3]. The Rel/NF-κB family of inducible transcription factors mediates cellular response to a broad array of stimuli by regulating the expression of hundreds of genes (enhancer sequences) with distinct functions, including immune, inflammatory response, programmable cell death, growth, proliferation, and development. These DNA sequences are collectively known as κB DNA sequences. In mammals, the Rel/NF-κB dimers arise from five polypeptides, p50 [4], p52, p65 [5], cRel, and RelB. The most abundant of these dimers are the p50/p65 heterodimer and the p50 homodimer. The existences of some, but not all, of the other possible dimers have been shown to exist in cells. The NF-κB family can be divided into two subgroups based on the presence or absence of an activation domain. The p50 and p52 do not contain a distinct activation domain and belong to class I. The other three members constitute the class II subfamily. It is generally believed that the homodimers of p50 and p52 and the p50/p52 heterodimer function as transcriptional repressors. The remaining combinations of dimeric NF-κB proteins, containing at least one monomer of p65, cRel, or RelB, act as activators. Rel/NF κB proteins share a region that shows over 45% sequence similarity across the entire family. This region, known as the Rel homology region (RHR), is responsible for DNA binding and subunit dimerization. Rel/NF-κB proteins also share similar structures. Most of the RHR is folded into two immunoglobulin-like domains connected by a 10-amino-acid linker; the N-terminal domain confers sequence specificity in DNA binding and the C-terminal domain is involved in dimerization as well as DNA backbone recognition. These structures show that, unlike most other transcription factors, NF-κB dimers do not use any secondary structure for contacting DNA. All the DNA contacting residues emanate from loops connecting secondary structures. Crystal structures of these complexes suggest that in their free form the N-terminal domains should be flexible concerning the dimerization domain [6,7,8].

There are several significant challenges to understanding the dynamic regulation of cellular signaling quantitatively due to its inherent complexity. The complexity is often beyond intuition alone, and the dynamic cannot be interpreted qualitatively in many cases. A large amount of time-resolved measurements of the circuit elements are required to develop a quantitative understanding of transcription regulation. Traditional approaches are usually not suitable for large-scale quantitative measurements. Common transcription factor assays, e.g., electrophoretic mobility shift assay (EMSA) and luciferase/GFP-based reporter, are often time/labor-intensive, time delayed due to gene expression (reporter gene), and inconsistent due to the perturbation of the equilibrium dynamic during separation (EMSA) [9]. More importantly, the strengths of the transcription factor activity are usually described qualitatively, which limits the development of a quantitative model of the underlying cellular circuitries. These shortcomings are mainly due to the lack of biosensors that can provide rapid, high-throughput, and quantitative measurements.

Molecular recognition and transduction mechanisms are two crucial elements in the design of molecular biosensors. Molecular recognition is typically achieved by the binding of targets with specific probes such as complementary oligonucleotides, and transductions are made possible by conjugating the probing molecule or a secondary probe with a radioactive, enzymatic, or fluorescent label [10]. DNA nanostructures provide a reliable and predictable scaffold for precisely positioning fluorescent dyes to form energy transfer cascades, which are called fluorescence resonance energy transfer (FRET) [11]. FRET is a mechanism describing energy transfer between two chromophores, the donor of which, initially in its electronic excited state, may transfer energy to an acceptor chromophore through a nonradiative dipole-dipole coupling. Bimolecular fluorescence complementation (BiFC)-based FRET (BiFC-FRET) assay was developed and widely used for visualization and identification of ternary complexes in living cells with a conventional three-filter FRET setup [12]. 

Recently, molecular beacons have been developed for the rapid detection of nucleic acids. The molecular beacon is an oligonucleotide probe that has a stem-and-loop structure and will undergo a spontaneous fluorogenic conformational change upon hybridization to the complementary nucleic acid target. The design provides a mechanism for both specific recognition and transductions of the hybridization events in a single step. The technique has been applied to various biological studies. 

The detection method employed here centers around a pair of single-stranded, complementary, oligonucleotide probes. One of these probes is complementary to the target sequence. Each probe has a modification: the fluorophore probe is labeled with a fluorophore on the 5′ end, and the quencher probe is labeled with a quencher molecule on the 3′ end [13]. In the absence of a target, the probes hybridize and bring the fluorophore and quencher in close proximity, dampening out the fluorescent signal. In the presence of a target, the probe with high affinity to the target is thermodynamically driven to unzip and bind with the target. This separates the fluorophore and quencher, thus allowing the fluorescence signal to be detected. 

This technique was used to detect the proteins [14], including NF-κB [15,16,17] and Nrf2 [18]. The FRET-based DNA technique can detect the active form of NF-κB protein with 90% detection efficiency, and the system is stable and highly regenerable [16]. This method is as sensitive as but much less labor-intensive than the widely-used electrophoretic mobility shift assay (EMSA) for measuring NF-κB DNA binding activities [15]. It can detect protein–DNA interaction quantitatively and specifically using a simple procedure: mix and measure [19]. Also, this FRET-based assay can be easily adapted for high-throughput screening of NF-κB activation. However, the previous methods do not consider the negative effects from the background noise and non-uniform initial fluorophore conditions.

A novel molecular binding scheme is provided with two specific contributions. (1) One normalization equation is created to remove the negative effects from the various initial fluorophore concentrations and the background noise; (2) a periodic shaking at a frequency can help to stabilize the DNA–protein binding. This novel molecular binding scheme based on vigorous shaking for rapid detection of NF-κB was demonstrated using purified p50 and p65 (NF-κB). The limit of detection is on the order of nanomolar. The molecular binding scheme allows rapid quantifications of DNA-binding proteins without any separation or immobilization step, which can easily be incorporated into an automated microfluidic system for high-throughput drug screening and large-scale interrogation of signaling networks [20,21]. The NF-κB biosensor takes advantage of the specific interaction between a DNA-binding protein and its corresponding binding sequence. In this molecular design, a double-stranded DNA probe is designed based on the binding sequence. The probe is labeled with a fluorophore and a quencher. A complementary single-stranded DNA competitor is also synthesized. In the absence of the target DNA binding protein, the competitor separates the fluorophore and the quencher, and an increased fluorescence intensity can be detected. In the presence of the protein, the molecular binding stabilizes the double-stranded DNA probe. The amount of the stabilized fluorescent probes should correlate to the amount of DNA binding proteins. Therefore, the concentration of the target molecule can be measured quantitatively based on the fluorescent intensity. The DNA–protein binding specificities were quantified by using oligonucleotide mass tags and mass spectroscopy [22], but the molecular-binding scheme provides a practical approach for rapid detection of NF-κB. 

## 2. Material and Methods

Groups of experiments are designed for the analysis of protein-DNA binding, including the calibration experiment of purified NF-κB p50; the Ig-κB DNA specificity experiment for homodimer p50, p65, heterodimer p50/p65, and BSA; and the nuclear extract experiment for TNF-α stimulated and non-TNF stimulated HeLa nuclear extracts.

### 2.1. Materials

The experiment of DNA pair biosensor for quantization of Nuclear Factor Kappa B is designed using an 80 μL well on a corning 384-well plate (Corning Inc., Corning, NY, USA), where the small well needs better mixing during the reaction process. The method of mixing the protein solution is to mix the solution by 5 min of vigorous shaking and 60 min of slow shaking.

#### 2.1.1. Probe Design

One set of the probe was designed to evaluate the molecular binding scheme. A double-stranded DNA probe was chosen according to the NF-κB binding sequence (-GGGACTTTCC-), which is the immunoglobulin light chain κ gene from HIV-LTR (Ig-κB). Two bases were added to avoid interference between the NF-κB protein and the fluorophore-quencher pair. Modification of the remaining sequence can adjust the equilibrium binding affinity of the probe. Fluorescein (6-FAM) and Iowa Black FQ were selected as the fluorophore-quencher pair. Integrated DNA Technologies Inc. synthesized DNA probes. Quantitative fluorescence measurements were taken in 384-well plates using a microplate reader (BioTek, Synergy 2, Winooski, VT, USA).

**Figure 1:**
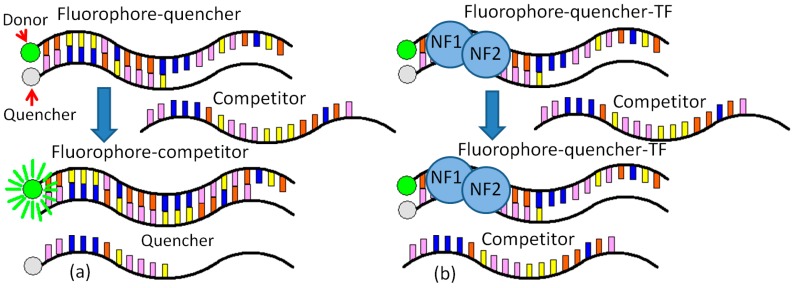
(**a**) Schematic of the fluorophore-conjugated probe is thermodynamically driven to switch between the quencher and the target. The DNA sequence on the dye is 5′ Fluorophore-TTGGGA CTTTCCCAAGATAGTAAG 3′, the quencher is 3′ Iowa black FQ-AACCCTGAAAGGGTTC 5′, and the competitor is 3′ AACCCTGAAAGGGTTCTATCATTC; (**b**) schematic of the sensing scheme for homogeneous detection of DNA binding proteins.

In the NF-κB experiment, the NF-κB probes are dye (5′ Fluorophore-TTGGGACTTTCC CAAGATAGTAAG 3′, Integrated DNA Technologies, Inc., San Diego, CA, USA) and quencher (3′ Iowa black FQ-AACCCTGAAAGGGTTC 5′, Integrated DNA Technologies, Inc.), while the NF-κB competitor is 3′ AACCCTGAAAGGGTTCTATCATTC 5′ (Integrated DNA Technologies, Inc.). In the final solution, there is a 20 nM dye, a 60 nM Quencher, and a 30 nM target (dye:quencher:target = 1:3:1.5). The target has a longer binding sequence with the dye than the quencher. The binding between the dye and the target is stronger than that between the dye and quencher (Figure 1a).

#### 2.1.2. Dye-Quencher Solution

After the dye and quencher are added into a 1.5 mL centrifuge tube, the deionized water, NaCl, and 1 M Tris-HCl buffer (0.1 M EDTA) are added to form 200 nM dye-quencher (300 nM dye, 900 nM Quencher, dye:quencher = 1:3) in buffer 10 mM Tris HCl and 100 nM NaCl. Then the solution is mixed by 1 min of vigorous shaking and 0.5 h of slow shaking.

After the dye-quencher solution is incubated for 5 min at 95 °C, the incubator is turned off and left to cool down to room temperature for about 3 h.

#### 2.1.3. NF-κB Protein

After the original Purified NF-κB p50 or p65 solution (Active Motif Co., Carlsbad, CA, USA) is centrifuged in the tube for 1 min, the solution is mixed with protein binding buffer (p50 dilution buffer:DTT = 200:1) to obtain 800 nM NF-κB protein. The solution needs to be vigorously shaken for 5 min and slow shaken for 0.5 h before being stored in −80 °C.

#### 2.1.4. Binding Buffer

1 M of Tris-HCl buffer (0.1 M EDTA), NaCl, MgCl_2_, 1 M DTT, and deionized water are mixed together to form a binding buffer with 10 mM Tris-HCl, 50 mM NaCl, 3 mM MgCl_2_, and 0.5 mM DTT.

#### 2.1.5. Protein Dilution buffer

The p50 dilution buffer (20 nM Tris-HCl, 200 nM NaCl, and 10% glycerol, bought from the Active Motif Co.) and 1 M DTT are mixed at ratio of 200:1 into a 1.5 mL centrifuge tube [23,24], and then the solution is served by 6 min of vigorous shaking and 1 h of slow shaking.

#### 2.1.6. Dilution of NF-κB or Other Proteins

According to the design, different concentrations of NF-κB or other proteins are obtained by being diluted with protein dilution buffer, followed by being vigorously shaken for 3 min and slow shaken for 30 min.

In the calibration experiment, the concentration of protein NF-κB p50 ranges from 0 nM to 100 nM (with ratio p50:dye = 0–5). The specificity experiment investigates 50 nM p50, 50 nM p65, 25 nM p59/p65, and 5000 nM BSA. The nuclear extract experiment compares 10 μg/well TNF-α stimulated HeLa Nuclear Extract and 10 μg/well 4-h Serum HeLa Nuclear Extract.

### 2.2. Denoising Methods

The deionized water is used to clean the centrifuge tubes, pipette tips, microplate wells, and the head of the pipette pump before use, because any particles from the air and other dirty things accumulated on the surface of tubes, wells and pipettes will destroy the whole experiment.

A fluorescence reader is used to selecting the high-quality wells by checking the clean wells on a 384-well plate to exclude contaminated wells. The method is to inject 80 μL of de-ionized water into the selected wells, followed by applying 2 min of various shaking and removing the water to clean the selected wells. A judgment can be made on which wells are indeed contaminated so that the noise from the well can be minimized.

As protein is very expensive, it is a good idea to do an experiment accurately by choosing more than the required number of wells before adding protein. After the binding buffer is added, the best-required number of wells are chosen by ignoring the wells with the lowest or highest fluorescence value, to make sure each well has almost the same condition.

### 2.3. Experiment Procedure for NF-κB Detection

The binding buffer (10 mM Tris-HCl, 50 mM NaCl, 3 mM MgCl2, 0.5 mM DTT) and protein dilution buffer are both required for the NF-κB experiments. The fluorescence data are obtained from the microplate reader after each shaking and are further normalized for FRET analysis. The detailed procedure for NF-κB Detection is provided as follows (Figure 1b).

1. Before the experiment, the probes are incubated in water at 95 °C for 5 min and slowly cooled down to room temperature for 2.5 h.
2. High-quality wells with low background fluorescence value are selected using the fluorescence reader and are cleaned by deionized water and variable shaking for 1 min.
3. The same amount of binding buffer is added to each well. The solutions are mixed by 20 s of variable shaking per minute for 10 min.
4. The solutions of NF-κB or other proteins were added to each well and mixed by 20 s of variable shaking per minute for 20 min.
5. The 20 nM dye-quencher is added to each well, and the reaction between the protein and the dye-quencher is treated for 2.5 h by variably shaking for 20 s per minute.
6. The 30 nM target is added to each well, and the reaction between the target and (dye-quencher)-p50 is treated for 5 h by variable shaking for 20 s every minute.

### 2.4. FRET Analysis

**Figure 2:**
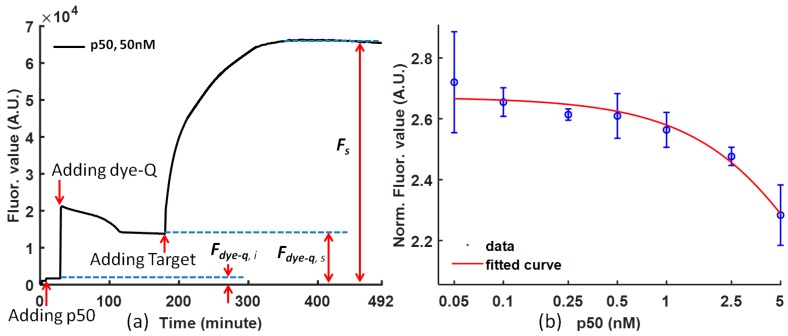
(**a**) The fluorescence value normalization method for the data of the NF-κB p50 experiment. NF-κB p50 was added to the 96-well microplate at time = 9, 20 nM dye-quencher was mixed at time = 30, and the target was added at time = 181. The solutions were mixed by medium shaking 3 s/5 min. Binding buffer was 10 mM Tris-HCl, 150 mM NaCl, and 2 mM MgCl_2_; (**b**) the decrease of fluorescence value with increased p50 concentration demonstrates that the Ig-κB binding sites on NF-κB p50 can bind with -GGGACTTTCC- DNA sequence, and inhibit the DNA competitor to separate the dye-quencher. The concentration ratios of p50 over 20 nM dye are 5, 2.5, 1, 0.5, 0.25, 0.1, and 0.05. The corresponding p50 concentrations are 50 nM, 20 nM, 10 nM, 5 nM, 2 nM, and 1 nM. The ratio of dye:quencher:target is 1:3:1.5 with 20 nM dye, 60 nM quencher, and 30 nM target, respectively.

In the FRET analysis, the following Equation (1) is used to normalize the data to reduce the influence of noise (Figure 2a).

(1) $$\mathrm{Normalized}\;\mathrm{FRET}\;=\;\frac{F_{s}-F_{dye-q,s}}{F_{dye-q,s}-F_{dye-q,i}},$$

where *F_s_* is the fluorescence signal intensity of the FRET probe bound by protein and competed by the target probe; *F_dye-q,s_* is the fluorescence signal intensity of the FRET probe and protein in steady status before adding target; *F_dye-q,i_* is the fluorescence signal intensity of the protein before adding dye-quencher; and *F_dye-q,s_* − *F_dye-q,i_* is the fluorescence signal intensity from the background.

The Equation (1) shows how to calculate the normalized fluorescence value from the original experimental data. As a result, the normalized FRET signal is the signal-to-noise ratio of the competitive reaction. This normalization considers the negative effects of the various initial fluorophore concentration and background noise so that it can be used to compare the molecular binding performance under the influence of different proteins.

### 2.5. Specificity Experiment

The Ig-κB specificity experiment is to analyze the binding of Ig-κB DNA with three different NF-κB dimmers: p50 homodimer, p65 homodimer, and p50/p65 heterodimer. Homo- and heterodimers of members of the Rel/NF-κB family specifically recognize the -GGGACTTTCC- nucleotide sequence. The p50/p65 heterodimers and the p50 homodimers are the most common dimers found in the NFκB signaling pathway. 

In this experiment, (1) the binding buffer solution consists of 10 mM Tris-HCl, 50 mM NaCl, 3 mM MgCl_2_, and 0.5 mM DTT; (2) 384-well microplate is used; and (3) the ratio of dye:quencher:target = 1:3:1.5. Several proteins, p50, p65 and BSA (albumin of bovine serum) are available to verify the specificity of Ig-κB from HIV LTR.

### 2.6. Nuclear Extracts Experiment

Two kinds of nuclear extracts, HeLa nuclear extract (4-h serum response, with positive transcription factors: c-Fos, Sp1, and SRF) and HeLa nuclear extract (TNF-α stimulated, with positive transcription factor: NF-κB), can be bought from Active Motif Company. To obtain the nuclear extract (4-h serum response), cells are cultured for 24 h in low serum (0.5%) conditions and then serum-stimulated (10%) for 4 h prior to harvesting, and this nuclear extract is supplied in dilution buffer (20 mM Hepes (pH 7.9), 100 mM KCl, 1 mM MgCl_2_, 20% glycerol, 0.5 mM PMSF and 0.5 mM DTT). The HeLa nuclear extract (TNF-α stimulated) is collected in Lysis Buffer (consists of 20 mM Hepes pH 7.5, 350 mM NaCl, 20% glycerol, 1% Igepal-CA630, 1 mM MgCl_2_, 0.5 mM EDTA and 0.1 mM EGTA) after a 30-min incubation with TNF-α (20 ng/mL). 

The nuclear extract experiment, designed on a 384-well microplate, uses a binding buffer solution composed of 10 mM Tris-HCl, 50 mM NaCl, 3 mM MgCl_2_, and 0.5 mM DTT. The ratio of dye: quencher: target is 1:3:1.5.

## 3. Results

Several strategies are taken to reduce the background noise for 384-well plates: (1) deionized water is used to clean the wells before use; (2) deionized water is also used to clean pipette tips and centrifuge tubes before use; and (3) the solution is mixed homogeneously. 

### 3.1. Quencher Characterization

Before performing a DNA detection experiment, the fluorophore and quencher probes were initially prepared in stock solutions. The probes were hybridized before mixing with target samples. An experimental procedure had been designed and tested to obtain maximal initial hybridization between the fluorophore and quencher probes. As incubation temperatures increase, the quencher probes are hybridized to the fluorophore probe, quenching the fluorescent signal at an increasing rate. Slow cooling down from an initial temperature of 95 °C is the most effective scheme, and, after an hour, the fluorescence value of the hybridized probes has been reduced to less than 10% of the initial value, yet cooling down from the room temperature reduces the fluorescence value to 50% of the initial value. Based on these results, all the fluorophore and quencher probes in experiments were performed according to this protocol.

In the molecular probe biosensor (MPB) design, the concentration of the quencher probe relative to that of the fluorophore probe can be flexibly adjusted to maximize the signal-to-noise ratio. This flexibility strategy represents one of the advantages of the MPB over other related molecular binding schemes. Firstly, the experiments were performed to determine the optimal quencher-to-fluorophore ratio [13]. The resulting data show that 3:1 ratio was able to quench fluorescence to a low noise level effectively.

### 3.2. Calibration Experiment

To evaluate and optimize the performance of the molecular assay, experiments were performed using purified p50 proteins (NF-κB). NF-κB was chosen based on its relatively well-known structure, regulation, function and its importance in combating inflammation. In the experiment, the probe was incubated with the protein for approximately 2.5 h. The competitor probe was then introduced into the well. The fluorescence intensities were monitored as a function of time after the addition of the protein and the competitor for over 5 h. 

The curves drawn from the raw experimental data are convenient for analysis, and it is necessary to map the experiment data to reduce the noise from the difference of the dye-quencher and the target for various solutions. The fluorescence value is normalized using Equation (1), and the p50 function can be clearly concluded from the tendency of the curve at steady status. Figure 2a is the temporal response of (dye-quencher)-p50 solution after adding the DNA competitor (the target) at time = 0 for representative p50. After the protein was mixed with the probe, a small decrease in fluorescent intensity was observed. It is likely that the p50 protein stabilizes the probe and reduces the fluorescent intensity. Upon the addition of the competitor DNA, the fluorescent intensity increased rapidly, which indicated a separation of the fluorophore and quencher pair. Figure 2b shows the effect of the concentration of p50 (with a concentration ratio over 20 nM dye at 5, 2.5, 1, 0.5, 0.25, 0.1, and 0.05) on the fluorescence intensity. Concentration dependence of the p50 protein on the fluorescence response was observed. The limit of detection is in the nanomolar range. The fluorescence intensity decreases with increasing concentration of p50. Therefore, the concentration of p50 can be determined based on the fluorescence intensity. Figure 2b also demonstrates the calibration of NF-κB p50 and Ig-κB DNA sequence binding. The higher the p50 concentration is, the more difficult it is for the DNA competitor to from separate the dye-quencher binding.

### 3.3. Specificity Experiment

**Figure 3:**
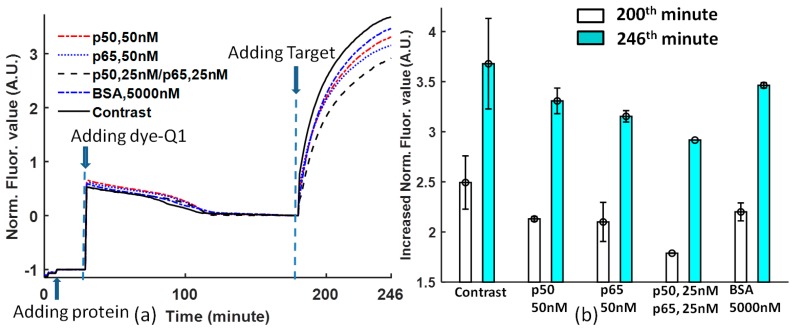
(**a**) The process of the Ig-κB specificity experiment. The protein was added to the tube at time = 9th minute; the dye-quencher was added to the protein solution at time = 30th minute; and the target DNA was added at the time = 181st minute. Four kinds of proteins were provided for specificity comparison: 50 nM p50, 50 nM p65, 25 nM p50/p65 (25 nM p50 + 25 nM p65), and 5000 nM BSA. The assay without protein was also provided for reference; (**b**) the comparison of protein DNA binding affinity to the Ig-κB DNA sequence for solutions with non-protein, 50 nM p50, 50 nM p65, 25 nM p50/p65, and 5000 nM BSA, respectively. The normalized data is obtained at the 200th and 246th minutes from Figure 3a.

Figure 3b is obtained at the 200th and 246th minutes from Figure 3a. The comparison of protein DNA binding affinity for solutions with non-protein, 50 nM p50, 50 nM p65, 25 nM p50/p65, and 5000 nM BSA shows that the DNA sequence -GGGACTTTCC- is selective to different proteins. p65 has higher affinity than p50 [25]; p50/p65 heterodimer has the highest affinity to Ig-κB DNA [26,27]; the Ig-κB DNA sequence is not sensitive to BSA. However, there is one argument about the affinity of the heterodimer and homodimer of p50 and p65. In Reference [25], p65 > p50/p65 > p50, but in References [26,27], p50/p65 > p50 > p65. In our experiment, the priority is p50/p65 > p65 > p50. 

The contrast that does not contain proteins has a high error, while the solutions with proteins will stabilize the DNA probe binding. 

### 3.4. Nuclear Extracts Experiment

**Figure 4:**
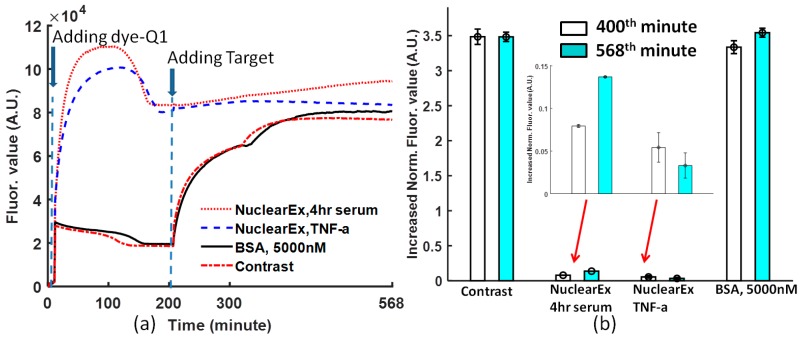
(**a**) The process of the nuclear extract experiment for 10 μg/well TNF-α–stimulated HeLa Nuclear Extract and 10 μg/well 4-h Serum HeLa Nuclear Extract. The dye-quencher was added to the protein solution at time = 0, and the target DNA was added at time = 197th minute; (**b**) comparison of increased normalized fluorescence values obtained at the 400th and 568th minutes from Figure 4a, after adding target DNA for 10 μg/well TNF-α–stimulated HeLa Nuclear Extract and 10 μg/well 4-h Serum HeLa Nuclear Extract. The data from Figure 4a are normalized following Equation (1).

From Figure 4a, when the dye-quencher was added to the protein and binding buffer solution, in the nuclear extract solutions, most of the dye-quencher double strands were dissociated, and the fluorescence value converged at one value higher than that of BSA and the non-protein solution. However, the specificity of the Ig-κB DNA can be detected if the target DNA was added to the solutions. The data obtained at the 400th and 568th minutes from Figure 4a are normalized, following Equation (1). In Figure 4b, the normalized value is 0.137 with a deviation of 0.0006 for nuclear Ex, 4-h serum, and 0.033 with a deviation of 0.014 for nuclear Ex, TNF-a. From Figure 4b, non-TNF–stimulated HeLa nuclear extract increased by a larger fluorescence value than TNF-α stimulated HeLa nuclear extract. Therefore, this biosensor is specific to the Ig-κB DNA sequence.

Even though the solution conditions are different, the created normalization Equation (1) is powerful enough to compare the molecular binding performance by removing the negative effect of the various initial fluorophore concentration and background noise.

The periodic shaking at a frequency is a specific feature of this protocol. If the shaking is stopped for one period, the curve will lose the coherent tendency just like in the case of Figure 4a. At the 320th minute, one short period of pause without shaking leads to the change of the binding condition.

### 3.5. “Convergence” and “Jump” Phenomena

There is a “convergence” phenomenon for each curve after the dye-quencher solution is added to the protein solution (Figure 2a). The reason is that the reaction is composed of two dynamic processes: one is the dissociation caused by glycerol (10%), the other one is the convergence caused by the dye and quencher probes under the influence of p50. In the nuclear extract experiment, the dissociation of dye-quencher is more severe (Figure 4a), because of more glycerol (20%) in the nuclear extract and an assumption that there are a few free DNA segments complementary to Ig-κB sites in the nuclear extract.

Similarly, there is a “jump” for each curve after adding target DNA (in Figure 2a, Figure 3a, and Figure 4a), the reason is that the dissociation reaction of Dye-Quencher is composed of two dynamic processes: One is the dissociation caused by glycerol, the other one is the dissociation caused by the competitive Target DNA. The different time constants of these two processes cause this “jump” phenomenon. If a 96-well microplate without using glycerol is chosen, and the solution is mixed by shaking at one frequency, this phenomenon will not occur (Figure 3a).

The time-limiting step of the MPB is the switching of binding partners. In other words, the total assay process time is primarily determined by the switching time. The kinetics of molecular switching at different temperatures and quencher lengths were measured to estimate the assay time. Molecular switching occurs in less than 2 min, which is at least one order of magnitude faster than conventional approaches for gene expression analysis. The fast switching time (i.e., the total assay time) of the MPB is advantageous for point-of-care diagnosis.

## 4. Discussion

The experimental data demonstrated the feasibility of the molecular probe for homogeneous detection of DNA binding proteins. Several aspects can be modified to enhance the performance of the assay. The assay time depends on the kinetics of the molecular switching, which is controlled by the affinity of the Ig-κB probe (-GGGACTTTCC-). Currently, the assay can be finished in approximately 5 h on a 384-well microplate. It is likely that a shorter probe sequence can further reduce the assay time. 

### 4.1. Functions of Dye-Quencher Fluorophore Probes and Competitive DNA Target

The functions of the dye-quencher fluorophore probes and the competitive DNA target are studied in detail by examining several key parameters affecting the performance of the assay. Several treatments of dye-quencher binding were performed, showing that heating at 95 °C for 10 min then cooling down to room temperature in water can achieve the best performance of dye-quencher binding in a short time. The design of the experiment can help us to determine the best ratio of quencher:dye, and with the ratio of 3:1, almost 90% of the oligonucleotide dye and quencher are bound together. The calibration experiment of the target DNA shows that this molecular probe biosensor has a detection limit on the order of nanomolar, and is capable of quantifying the concentration of specific nucleic acid targets in less than 2 min. It is also proved that a more extended DNA target is more competitive than a shorter one.

The molecular binding scheme was demonstrated by a calibration experiment using purified p50 (NF-κB). In the dynamic process, the “convergence” and “jump” phenomena are caused by glycerol and well size. The calibration experiment of NF-κB p50 indicates that the detection limit is determined by the binding performance of dye and quencher oligonucleotides, and DNA-binding protein NF-κB stabilizes only a small portion of the probes. At equilibrium, a portion of the protein NF-κB (depending on the probe concentration) binds to the probe. Therefore, the probe concentration should be optimized such that the protein binding stabilizes a larger portion of the probes. The saturation of dye-quencher binding determines the detection limit. Fortunately, the dye-quencher binding performance can be improved by annealing from 95 °C, avoiding secondary structure of quencher, a high ratio of quencher:dye of about 3:1, a shorter DNA sequence, or other methods.

The specificity experiment of Ig-κB DNA shows that p50/p65 heterodimer has the highest affinity for Ig-κB DNA; p65 homodimer binds with intermediate affinity, whereas p50 shows the lowest binding affinity, and Ig-κB DNA is not sensitive to BSA (bovine albumin serum). Although there are arguments about the affinity property of NF-κB, this method can provide one convenient and persuasive way to make a better evaluation.

The experiment of HeLa nuclear extract shows that TNF-α stimulated HeLa nuclear extract has higher affinity to Ig-κB DNA than non-TNF stimulated HeLa nuclear extract (4-h serum response). Although there exists a strong dissociation activity of double-strand DNA dye-quencher, this experimental protocol can still stabilize the reaction to one converged value, so that the competitor target DNA can have a better performance.

### 4.2. Binding Affinity

The wild-type κB site exists in an equilibrium, being either inherently straight or bent 20° towards the major groove while the mutations, biochemically observed to abrogate protein binding, effect a pronounced kink that precludes protein binding [6]. Indeed, in vitro DNA-bending experiments show that the various κB sites do have inherently different bend angles. An intrinsic bend found in various κB sites dictates via indirect readout what type of protein conformation is required for binding and thus affects the resulting transcriptional activity [28].

Binding affinities are comparable for the three NF-κB/DNA complexes under test: p50/p65/DNA, p50 homodimer/DNA, and p65 homodimer/DNA complexes [29]. The nature of the binding isotherms indicates a cooperative mode of binding [27,30]. The source of cooperation is likely to be the stepwise binding of NF-κB monomers to DNA half-sites followed by subunit association through the dimerization domains of each protein subunit [31]. The investigation of binding by p50 to Ig-κB DNA clearly suggests that the dimer recognizes the target in a highly cooperative manner, and the primary source of the cooperativity is indeed the dimerization interactions between the two p50 subunits.

The cooperative model predicts that the monomers bind sequentially to their DNA half sites, with the second monomer binding to its half site with much higher affinity due to its interaction with the pre-bound first subunit.

### 4.3. pH, Salt, and Temperature

The role of pH, salt and temperature on the formation of the p50/p65 heterodimer/Ig-κB complex was characterized [22]. The heterodimer binds to the Ig-κB DNA target in a pH-dependent manner, with the highest affinity between pH 7.0 and 7.5. A strong salt-dependent interaction between Ig-κB and the p50/p65 heterodimer is observed, with optimum binding occurring at monovalent salt concentrations below 75mM, with binding becoming virtually nonspecific at a salt concentration of 200 mM. Binding of the heterodimer to DNA was unchanged across a temperature range from 4 °C to 42 °C.

The sensitivity to the ionic environment and insensitivity to the temperature indicate that NF-κB p50/p65 heterodimers form complexes with specific DNA in an entropically-driven manner.

### 4.4. Nuclear Extract

NF-κB p50 does not contain a distinct activation domain, while p65 contains an activation domain. The transcription factor NF-κB p65 (nuclear factor κB) exists in an inactive form bound to the inhibitory IκB proteins in the cytoplasm. Treatment of cells with various inducers such as lipopolysaccharide, TNF or IL-1, results in the degradation of the IκB proteins (See the Active Motif’s FunctionELISA™ IκBα Kit) [32,33]. This treatment releases NF-κB dimers, which subsequently translocate to the nucleus where they activate the appropriate target genes. 

## 5. Conclusions

In summary, the proof-of-principle of a molecular probe biosensor has been demonstrated for rapid detection of DNA-binding protein NF-κB. This selective molecular assay has high sensitivity and can be implemented in a high-throughput microplate format. Further optimization of the probe design and assay conditions will improve the signal-to-noise ratio and assay time of the molecular sensor. Since a gel separation or immobilization step is not required, the equilibrium binding kinetics of NF-κB-(dye-quencher) would not be disturbed as in EMSA. More importantly, the homogenous assay allows rapid, quantitative, high throughput, and automated measurements of the DNA-binding protein NF-κB at low cost, which is beneficial for automated drug screening systems. The rapid detection of the low abundance of nucleic acids has been demonstrated by incorporating molecular beacons and an ultrasensitive molecule detection system. In the future, the microfluidics MEMS devices will be designed with an automated process to be disposable with small sample volume and low cost, supporting simultaneous mRNA and transcription factor profiling.
